# Water drainage from Kushiro Coal Mine decreased on the day of all *M* ≥ 7.5 earthquakes and increased thereafter

**DOI:** 10.1038/s41598-018-34931-5

**Published:** 2018-11-07

**Authors:** Yoshiaki Fujii, Yoshihisa Ichihara, Hiroyuki Matsumoto, Jun-Ichi Kodama, Daisuke Fukuda, Anjula B. N. Dassanayake

**Affiliations:** 1 Rock Mechanics Laboratory, Hokkaido University, N13W8, Sapporo, 060-8628 Japan; 2 Kushiro Coal Mine, 5-2-23, Okotsu, Kushiro, 085-0081 Japan; 3 Department of Earth Resources Engineering, University of Moratuwa, Moratuwa, 10400 Sri Lanka

## Abstract

The amount of water drainage from Kushiro Coal Mine in Hokkaido, Japan decreased on the day of all *M* ≥ 7.5 earthquakes with epicenters within 300 km of the mine during the monitoring period and increased after these earthquakes. This is a valuable finding which would give us a clue to understand pre- and post-seismic rockmass behaviors and contribute for progress in earthquake prediction in future.

## Introduction

A number of reports have suggested the pre-seismic changes of groundwater level, but only a few recorded data of pre-seismic groundwater drops were published in peer-reviewed journals. For example, a month and a half before 1978 Izu-Oshima-Kinkai earthqauake (*M*7.0), a drop of groundwater level and temperature, at 30 km away from the Izu earthquake epicenter was reported^1^. Orihara *et al*.^2^ had reported that the water level and temperature at Goyo Hot Spa which is located 250 km from the epicenter of the Tohoku 2011 earthquake (*M*9.0), was dropped by 30 m and 2 °C respectively, 3 months before the earthquake and the water level was increased after the earthquake. The amount of water drainage from Kushiro Coal Mine in Hokkaido, Japan was monitored between January 1, 1993 to April 30, 2015 and it decreased on the day of all *M* ≥ 7.5 earthquakes with epicenters within 300 km from the mine and increased thereafter. This phenomenon is reported here because such phenomena may be important for understanding the changes in rock mass conditions before and after large earthquakes and might give clues to earthquake prediction together with the above case studies.

Kushiro Coal Mine is located on the coast of the Pacific Ocean, where the mining of Paleogene bituminous coal occurs under the ocean bottom (see Fujii *et al*.^3^, for details of the geology, mining method, etc.). Kushiro Coal Mine took over the operations of Taiheiyo Coal Mine on January 31, 2002. Therefore, data recorded by Taiheiyo Coal Mine are also presented. The water flowing into Taiheiyo Coal Mine was not seawater, but was fossil water from holes drilled in Cretaceous rock for water drainage and groundwater from the land^4^. The water flowing into Kushiro Coal Mine is only groundwater from the land.

**Figure 1:**
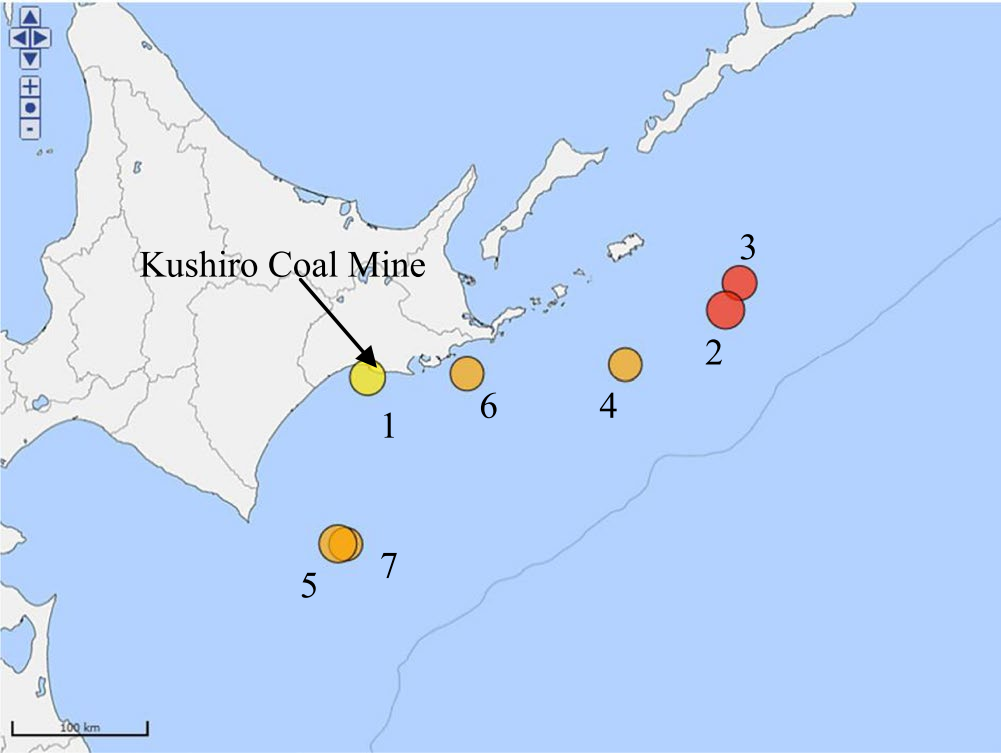
All *M* ≥ 7 earthquakes in the period of monitoring around Kushiro Coal Mine (source: Japan Meteorological Agency website, http://www.data.jma.go.jp/svd/eqdb/data/shindo/index.php). Yellow, orange and red denote focal depth > 100 km, 100–30 km and < 30 km respectively.

Water inflow itself was not measured, but the amount of daily water drainage from 7 am to 7 am JST (Japan Standard Time, UTC (Coordinated Universal Time) +9 hours) on the next day from the No. 2 pump pit (N42.96°, E144.41°, Fig. 1) located 152 m below sea level was recorded. The variation in water drainage can be roughly regarded as the variation in water inflow because only a small, relatively constant amount of collected water is used for machinery cooling and showers to prevent rock dust.

## Results

**Figure 2:**
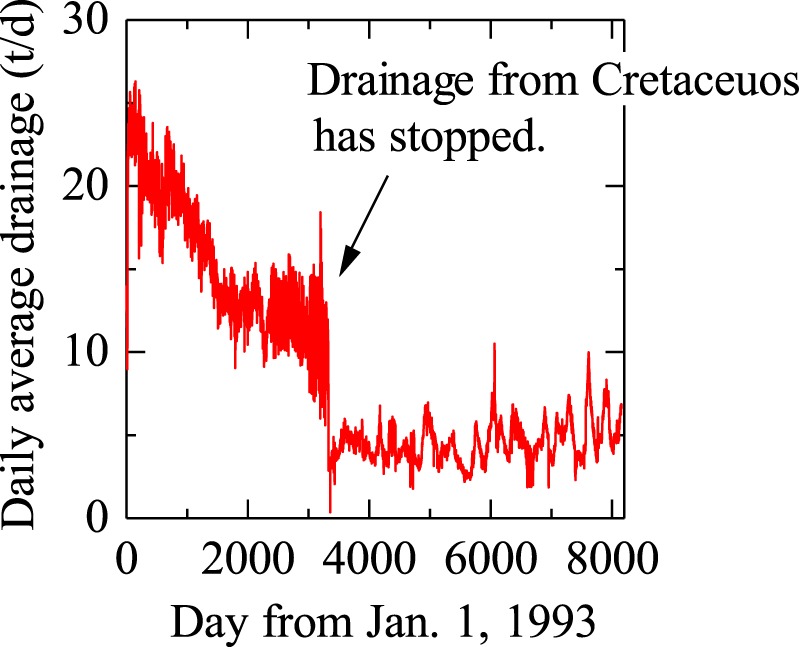
Daily average drainage for the whole monitoring period.

**Figure 3:**
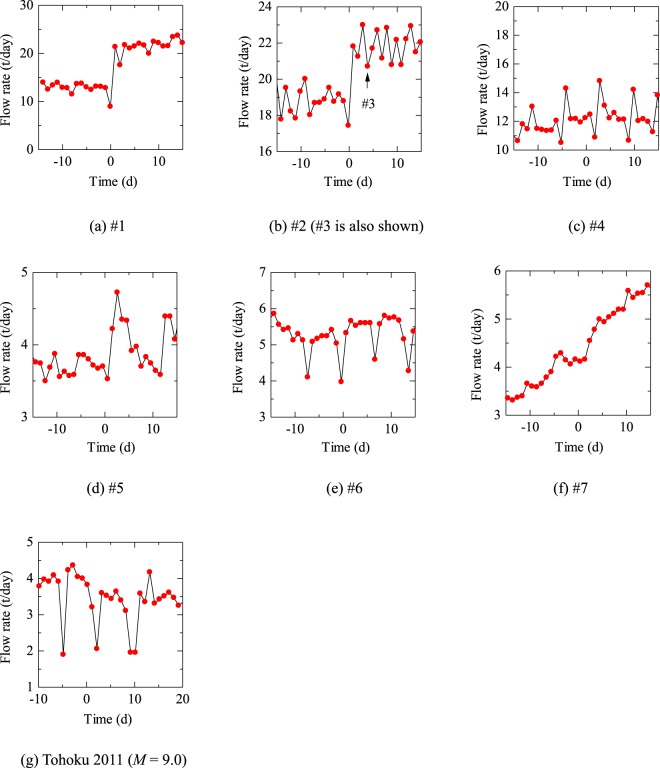
Variation in the amount of water drainage with each earthquake. The time when each earthquake occurred was set as time zero. Tohoku 2011 was centered far from Kushiro Coal Mine and is shown for comparison. The drainage amount was accumulated from 7:00 JST to 7:00 JST on the next day and regarded as the value at 19:00 JST. For example, in the case where an earthquake occurred on 17:30 JST, the datum will be plotted on 1.5/24 = 0.063 (days).

Seven *M* ≥ 7 earthquakes, including two giant (*M* ≥ 8) earthquakes, occurred during the monitoring period within 300 km from the coal mine and were catalogued in a database by the Japan Meteorological Agency (Fig. 1 and Table 1). The amount of water drainage during the entire monitoring period were shown in Fig. 2. It gradually decreased with time and, after the sudden decrease due to the abandonment of the drainage from Cretaceous, became almost constant. The amount of water drainage in the period of 15 days before and after all *M* ≥ 7 earthquakes were shown in Fig. 3. The regular variations in a 1-week period typically seen in (c), (e) and (g) occurred because some pumps in the mined out areas were shut down on Sunday for safety reasons and did not exhibit any variation in water inflow. The amount of water drainage is larger for Taiheiyo Coal Mine (Fig. 3a–c) than that for Kushiro Coal Mine (Fig. 3d–g). This is because Kushiro Coal Mine quit the drilling to Cretaceous thereby abandoned the deeper mining areas as stated above.

**Table 1:** All *M* ≥ 7 earthquakes near the Kushiro Coal Mine in the monitoring period (source: Japan Meteorological Agency website, http://www.data.jma.go.jp/svd/eqdb/data/shindo/index.php). Tohoku 2011 is shown for comparison.

Number	Time (JST)	Latitude (degrees)	Longitude (degrees)	Focal depth (km)	Magnitude	Epicentral/ hypocentral distance (km)
#1	1993/01/15 20:06:07.2	42.92	144.35	101	7.5	7/101
#2	1994/10/04 22:22:56.9	43.38	147.67	28	8.2	269/270
#3	1994/10/09 16:55:39.0	43.56	147.80	0	7.3	283/283
#4	2000/01/28 23:21:08.7	43.01	146.74	59	7.0	190/199
#5	2003/09/26 04:50:07.4	41.78	144.08	45	8.0	134/141
#6	2004/11/29 03:32:14.5	42.95	145.28	48	7.1	71/86
#7	2008/09/11 09:20:51.3	41.78	144.15	31	7.1	133/137
–	2011/03/11 14:46:18.1	38.10	142.86	24	9.0	557/558

It can be seen that the amount of the water drainage decreased on the day of earthquake and increased afterward for earthquakes #1 (Fig. 3a), #2 (Fig. 3b) and #5 (Fig. 3d) although the decreased and increased pattern was not obvious for #5. The pattern was checked (see Methods) for the entire monitoring period and only those for #1 and #2 were detected.

## Discussion

**Figure 4:**
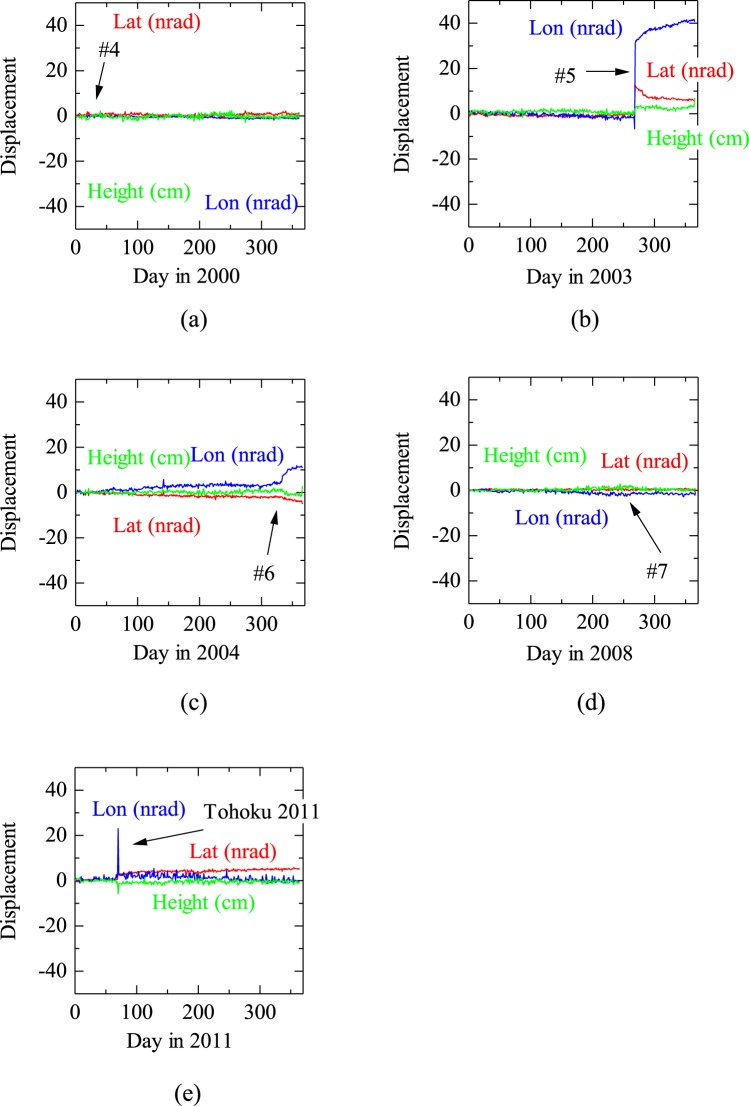
GPS solution for Kushiroshi GPS station at N42.96°, E144.43° (#940010).

The Geospatial Information Authority of Japan provides daily coordinates of GPS targets in Japan from 1996. Therefore, it is not possible to investigate the GPS results for the earthquakes #1–#3 (1993–1994). However, the change in daily coordinates for #4–#7 were shown in Fig. 4 and displacements due to the earthquakes were found for #5 (Fig. 4b), #6 (Fig. 4c) and Tohoku 2011 (Fig. 4e).

**Figure 5:**
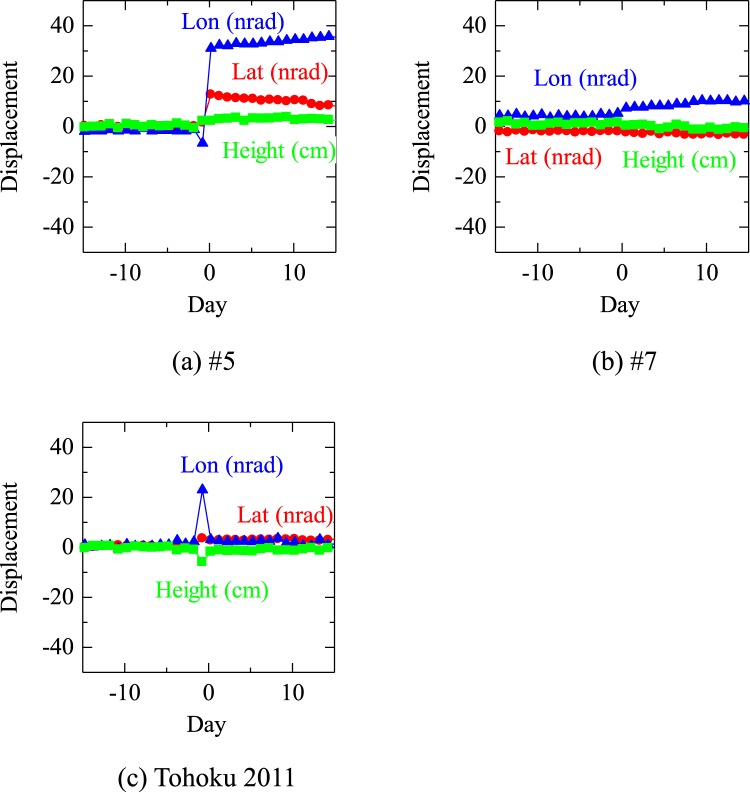
GPS solution for Kushiroshi GPS station 15 days before and after each earthquake. The GPS solutions are given as daily average values in UTC and regarded as the values at 12:00 UTC. For example, in the case where an earthquake occurred on 17:30 UTC, the datum will be plotted on −5.5/24 = −0.229 (days).

The displacements for #5 earthquake were clear and that in longitude shows an opposite motion, which might be related to the pre-seismic decrease in drainage, just before #5 (Fig. 5a).

For earthquake #7, even the largest permanent displacement in longitude was unclear (Fig. 5b). Displacements for Tohoku 2011 earthquake were mainly transient and far-field ones in which permanent displacements were very small. The above characteristics are in harmony with the drainage observation in which the decreased and increased pattern was not obvious but observed for #5 and that was not observed for #7 or Tohoku 2011 earthquakes.

**Figure 6:**
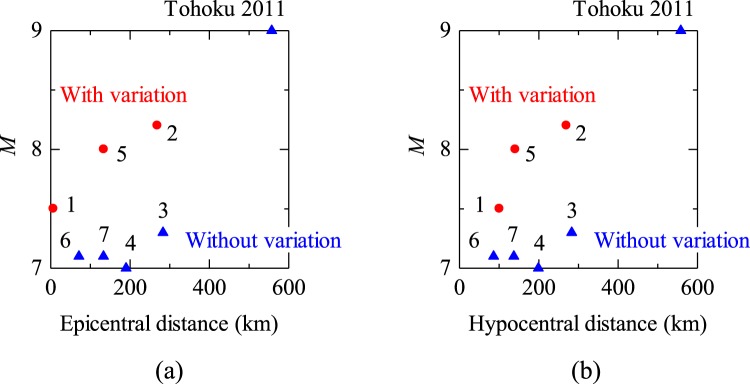
Effects of the magnitude and epicentral (**a**) and hypocentral (**b**) distances on the variation in the amount of drainage water.

The plots of epicentral or hypocentral distance versus magnitude (Fig. 6) imply that large earthquakes tend to have greater effects, although the Tohoku 2011 earthquake neither affected the amount of water drainage nor caused the permanent surface displacements because it was far from the mine. From the plot, more distinct decrease and increase pattern was expected for #5. The unclear pattern for #5 would be due to the decrease of the sensitivity of the drainage amount to the crustal deformation as a consequence of the abandonment of drainage from Cretaceous formation. It is estimated that the change in the drainage amount would have been mainly caused rather by the drainage of the fossil water from Cretaceous than the groundwater from the surface.

Orihara *et al*.^2^ postulated that the mechanism of the change in the water level and temperature at Goyo Hot Spa due to the Tohoku 2011 was a decrease in pore pressure due to dilatancy and the formation of new water paths as a result of slow slippage before the main shock. The cause of the change in the drainage amount from Kushiro Coal Mine cannot be clarified at this stage because the 1-day time resolution of the drainage data as well as the GPS solutions are not small enough and also more data including geological, geo-hydrological, geochemical etc. are needed. However, it is estimated that a decrease in groundwater pressure due to dilatancy before the earthquakes and an opening of discontinuities due to stress relief after the earthquakes would have played a significant role as a part of the mechanisms for the changes in the drainage.

The decrease in groundwater levels were observed a month and a half before Izu-Oshima-Kinkai earthquake^1^ and three months before Tohoku 2011earthquake^2^. It would be very difficult to predict when earthquakes will occur from those observations. On the other hand, the decreases in drainage at Kushiro Coal Mine began on the day of the each earthquake and it would rather be easier for immediate predictions of large earthquake occurrences.

There are many deep mines in the world and it is expected that they can work as much larger sensors for pre-seismic changes of rockmass conditions by recording the amount of drainage etc. without spending huge costs than newly drilling small diameter drill holes from the ground. The authors fully recognize that the pre-seismic decrease itself cannot be used to predict earthquake at this stage and hope that data accumulation from observations at a shorter sampling time at deeper mines will contribute to clarify the drainage pattern at large earthquakes and to develop earthquake prediction methods in future.

## Methods

### Detection of the decrease and increase pattern

**Figure 7:**
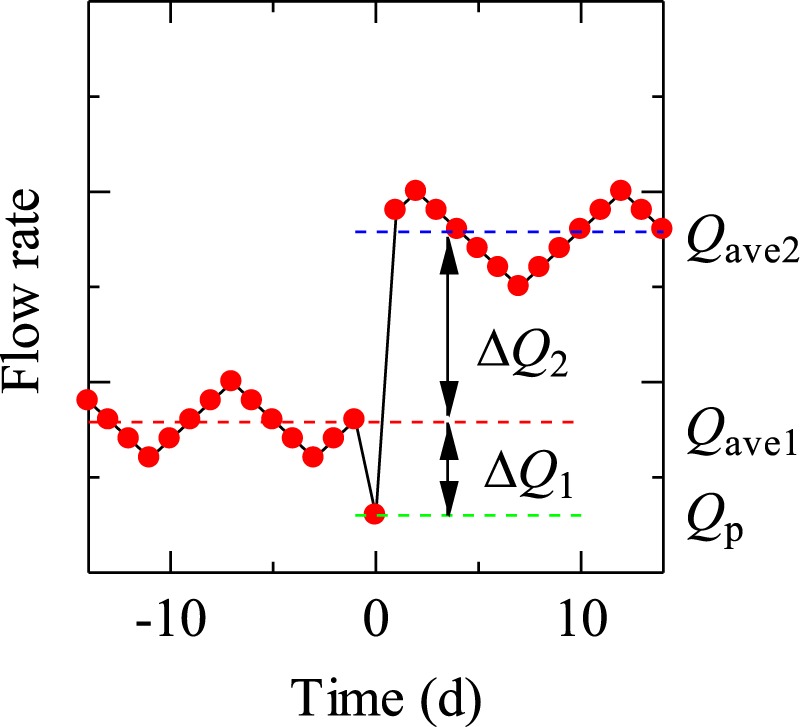
Schematic figure showing definitions of the variables to check the decrease and increase pattern.

Firstly, the average values *Q*_ave1_ and *Q*_ave2_ for the period 14 of days prior and succeeding a day are defined (Fig. 7). Then the pre-seismic drainage decrease Δ*Q*_1_ is defined as

$${\rm{\Delta }}{Q}_{{\rm{1}}}={Q}_{{\rm{p}}}-{Q}_{{\rm{ave1}}}$$

where *Q*_p_ is the drainage amount on the day. The post-seismic drainage increase Δ*Q*_2_ is defined as

$$\Delta {Q}_{{\rm{2}}}={Q}_{{\rm{ave2}}}-{Q}_{{\rm{ave1}}}.$$

**Figure 8:**
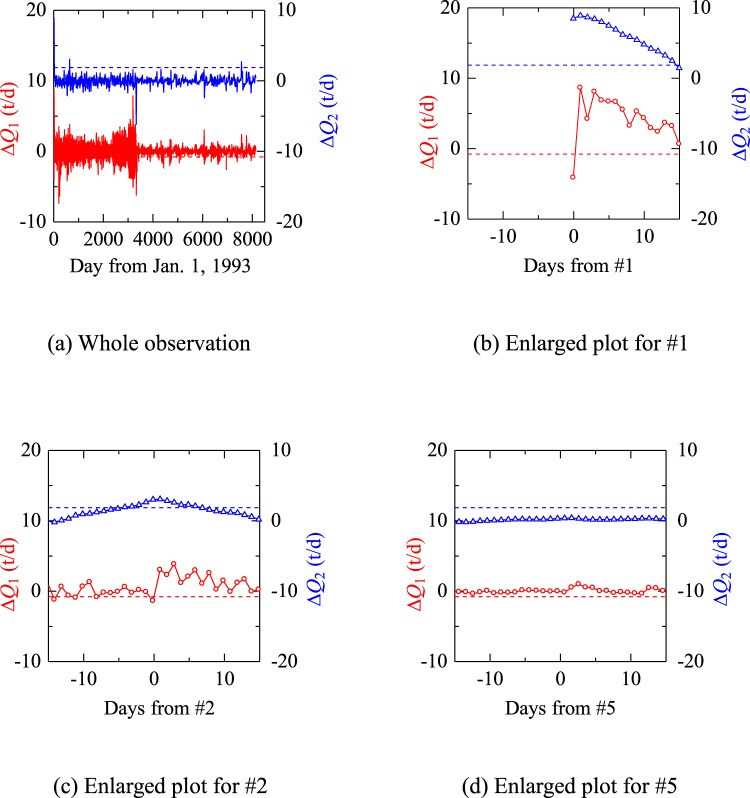
Pre- and post-seismic change in drainage Δ*Q*_1_ (the red solid line) and Δ*Q*_2_ (the blue solid line) with thresholds for the decrease (the red broken line) and increase (the blue broken line) pattern. #1 and #2 satisfies the both thresholds.

Δ*Q*_1_ and Δ*Q*_2_ were calculated for the each and every day in the monitoring period except for the first and last 14 days (Fig. 8), and the average values and standard deviations for Δ*Q*_1_ and Δ*Q*_2_ were calculated. The days which satisfied that they exceeded certain threshold values were selected as the days which showed the decrease and increase pattern. Setting the threshold values as the average value minus the standard deviation and the average value plus three times the standard deviation for Δ*Q*_1_ and Δ*Q*_2_, respectively, it was found that only the days for #1 (Fig. 8b) and #2 (Fig. 8c) satisfied the threshold values for the entire monitoring period. The pre-seismic decrease is obvious for #1 but not for #2. This would be mainly due to the too slow time resolution. Faster sampling, for example, at each hour of drainage amount is desired for better results.

## Data Availability

Earthquake database was from the website of the Japan Meteorological Agency (http://www.data.jma.go.jp/svd/eqdb/data/shindo/index.php). GPS solutions were downloaded from the Geospatial Information Authority of Japan (https://terras.gsi.go.jp/sso_login.php). The solutions were calculated using Bernese GNSS Software (http://www.bernese.unibe.ch) from the whole raw GPS data for each UTC day and given as daily values. They can be considered as average daily coordinates of the site.
